# SNAIL1: Linking Tumor Metastasis to Immune Evasion

**DOI:** 10.3389/fimmu.2021.724200

**Published:** 2021-11-30

**Authors:** Xiaolong Tang, Xue Sui, Liang Weng, Yongshuo Liu

**Affiliations:** ^1^ Department of Laboratory Medicine, Binzhou Medical University, Binzhou, China; ^2^ Department of Oncology, Xiangya Cancer Center, Xiangya Hospital, Central South University, Changsha, China; ^3^ Key Laboratory of Molecular Radiation Oncology Hunan Province, Xiangya Hospital, Central South University, Changsha, China; ^4^ Hunan International Science and Technology Collaboration Base of Precision Medicine for Cancer, Xiangya Hospital, Central South University, Changsha, China; ^5^ Hunan Provincial Clinical Research Center for Respiratory Diseases, Xiangya Hospital, Central South University, Changsha, China; ^6^ Institute of Gerontological Cancer Research, National Clinical Research Center for Gerontology, Changsha, China; ^7^ Center for Molecular Imaging of Central South University, Xiangya Hospital, Changsha, China; ^8^ Department of Clinical Laboratory, Binzhou Medical University Hospital, Binzhou, China; ^9^ Biomedical Pioneering Innovation Center (BIOPIC), Beijing Advanced Innovation Center for Genomics, Peking-Tsinghua Center for Life Sciences, Peking University Genome Editing Research Center, State Key Laboratory of Protein and Plant Gene Research, School of Life Sciences, Peking University, Beijing, China

**Keywords:** Snail1, EMT, signaling pathway, ubiquitination, methylation, acetylation, tumor immunity

## Abstract

The transcription factor Snail1, a key inducer of epithelial-mesenchymal transition (EMT), plays a critical role in tumor metastasis. Its stability is strictly controlled by multiple intracellular signal transduction pathways and the ubiquitin-proteasome system (UPS). Increasing evidence indicates that methylation and acetylation of Snail1 also affects tumor metastasis. More importantly, Snail1 is involved in tumor immunosuppression by inducing chemokines and immunosuppressive cells into the tumor microenvironment (TME). In addition, some immune checkpoints potentiate Snail1 expression, such as programmed death ligand 1 (PD-L1) and T cell immunoglobulin 3 (TIM-3). This mini review highlights the pathways and molecules involved in maintenance of Snail1 level and the significance of Snail1 in tumor immune evasion. Due to the crucial role of EMT in tumor metastasis and tumor immunosuppression, comprehensive understanding of Snail1 function may contribute to the development of novel therapeutics for cancer.

## Introduction

Metastasis is one of the most prominent features of malignant tumors and is the leading cause of death in tumor patients (1). Tumor metastasis is a multi-step process in which EMT has a crucial regulatory role. During the process of EMT, epithelial cells lose their cell polarity and cell–cell adhesion, and transit to quasi-mesenchymal cell states, thus increasing their migration and invasion properties (2). Recent studies indicated that tumor progression and metastasis are closely related to epigenetic modifications and the immune system. It was reported that immune checkpoint molecules such as PD-L1 are involved in EMT regulation, while EMT can also induce immunosuppression and immune evasion in tumors (3).

The Snail family of zinc finger transcription factors comprises three members in vertebrates, Snail1 (Snail), Snail2 (Slug), Snail3 (Smuc) (4, 5). Snail1 and Snail2 down-regulate the expression of many target proteins associated with EMT. Among them, the most significant one is E-cadherin (6). Due to the critical role of Snail1 in EMT, this mini review focuses on how appropriate Snail1 levels are maintained in cells, with emphasis on the role of epigenetic and UPS in the regulation of Snail1. Furthermore, we also discuss the involvement of Snail1 in tumor immune evasion, a role which has made it a promising therapeutics target in tumor treatment.

## Structural and Functional Characteristics of Snail1

**Figure 1:**
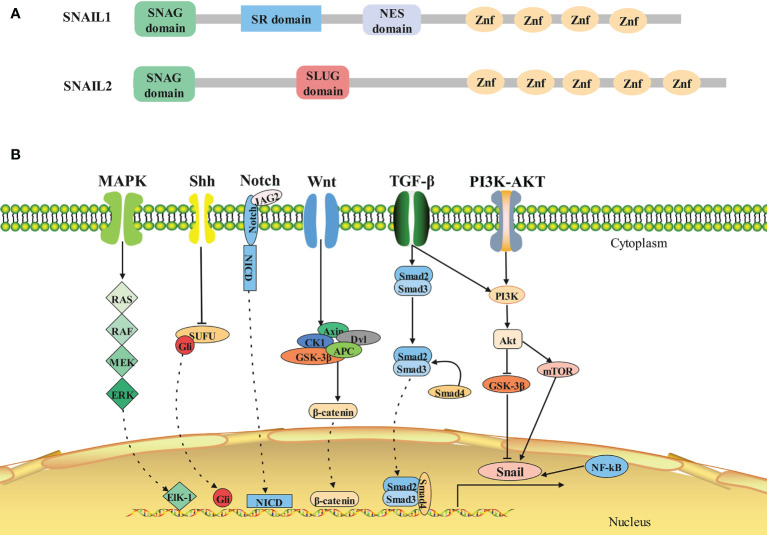
Structure and signaling pathways of SNAIL1. **(A)**Architecture of SNAIL1 in human. Composite of the overall structure of Snail1 and Snail2, which shows the relative positions of the SNAG domain, the zinc fingers (I–V), and the Slug-specific boxes, NES domain and serine-rich domain. **(B)**The molecular signaling pathways of SNAIL1. Snail1 is regulated by several signaling pathways that promote its expression. From left to right: MAPK, Shh, Notch, Wnt, TGF-β, PI3K-AKT, and NF-κB signaling pathway.

In all Snail family members, the amino terminal end contains a highly conserved SNAG domain, which functions as a transcriptional repressor domain (7). The fingers correspond to the C2H2 type and bind to the upstream regulatory region of target genes for gene specific transcriptional inhibition (8). The central region of Snail2 includes the Slug domain, while Snail1 has two defined functional domains in this region: a regulatory domain containing an Xpo1/CRM1 mediated nuclear export signal (NES) (9) and a serine-rich domain involved in the regulation of its stability (10) **(** **Figure 1A** **)**.

Snail1, as a transcriptional repressor, is implicated in the regulation of other tumor metastasis suppressors, such as the epithelial marker E-cadherin (11). Previous studies reported that SNAG domain of Snail1 couples on the *CDH1* (which encodes E-cadherin) promoter (12), and recruits histone deacetylase (HDAC). Subsequently, Snail1, HDAC1, HDAC2 and mSin3A conjointly form a multi-molecular complex that further inhibits the expression of E-cadherin (13). Furthermore, Snail1 interacts with the H3K9 methyltransferase G9A or SUV39H1 and recruits it to the *CDH1* promoter for transcriptional inhibition in breast cancer (14, 15), collectively resulting in the occurrence of EMT.

## The Signaling Pathways Involved in Snail1 Expression

The expression of Snail1 is regulated by many signaling pathways both at the transcriptional and protein level (16, 17) **(** **Figure 1B** **)**. Physiologically, these signaling pathways control normal cell morphology, proliferation, differentiation, and apoptosis. However, abnormal activation of these signaling pathways contribute to the initiation and progression of tumors activated (18, 19).

### TGF-β Pathway

The transforming growth factor-β (TGF-β) was described as an inducer of EMT during the development of tumor (20). Mechanistically, TGF-β binds to its receptor TβRII and TβRI, which subsequently phosphorylates its downstream targets, including members of the SMAD family of signal transducers, SMAD2 and SMAD3 (21), forming a heterooligomeric complex with SMAD4 (22). Then this SMAD complex translocates to the nucleus and functions as a transcription factor to regulate the transcription of target genes such as Snail1 in human tumors (16, 21, 23–25).

### PI3K-Akt Pathway

The phosphatidylinositol 3-kinase (PI3K)-Akt signaling pathway is hyperactivated or altered in many cancer types (26–28) and regulates a broad range of cellular processes (29, 30). It is well known that Akt can phosphorylate and inhibit GSK-3β activity, subsequently suppressing the GSK-3β-mediated phosphorylation of Snail1 and facilitating its stabilization and nuclear localization, which ultimately promotes EMT widely presenting in a variety of tumors (31–36). In addition to GSK-3β, PI3K-Akt also activates mTOR, thereby potentiating Snail1 expression in gastric, breast, pancreatic and ovarian cancer (37–40). Furthermore, some studies also indicate TGF-β regulates Snail1 expression *via* the Akt/GSK-3β signaling pathway in osteosarcoma and ovarian clear cell carcinoma (41, 42).

### Wnt Pathway

In the presence of Wnt signaling, the destruction complex (APC, Dvl, Axin, GSK-3β, CK1) reduces the phosphorylation and ubiquitination of β-catenin (43).Thus, levels of cytoplasmic β-catenin rise, which translocates to the nucleus and induces transcription of pro-invasive factors (44–46). Moreover, β-catenin/T-cell factor (TCF) transcriptional complex regulates Snail1 *via* Axin2-mediated nuclear export of GSK-3β in breast cancer (47).

### Notch Pathway

Notch signaling is generated through the interaction between Notch receptors and ligands such as Jagged-2 (JAG2) (48). Notch is released into the cytoplasm by intracellular segment (NICD) after being sheared three times (49), and then enters the nucleus to bind to the Snail1 promoter, directly stimulating transcription (50).

### Shh Pathway

Sonic Hedgehog (Shh) is a lipid-modified secreted protein that couples to Patched receptor (51). In the presence of Hedgehog signaling, Smoothened is relieved from Patched-mediated suppression due to the Hedgehog-dependent internalization of Patched, which leads to inactivation of SUFU for the stabilization and nuclear accumulation of Gli family members (51, 52). So far, Shh-mediated Gli1 activation was reported to induce the expression of Snail1 in a variety of cancers, such as breast, skin, ovarian, pancreatic, neuroendocrine cancer and basal cell carcinoma (45, 53–57).

### MAPK Pathway

The Ras/Raf/MEK/ERK pathway is the most important signaling cascade among all MAPK signal (58). Once activated, ERK translocates to the nucleus, binds to and regulates the activity of the transcription factor Elk-1 through phosphorylation (58). Of note, activation of Elk1 facilitates recruitment of phosphorylated mitogen and stress activated protein kinase 1 (MSK1), which in turn enhances histone H3 acetylation and phosphorylation (serine 10) of Snail1 promoter, ultimately promoting the transcription of Snail1 (59). Furthermore hepatocyte growth factor (HGF) could induce transcription of Snail1 by activating MAPK signaling pathway in liver cancer (60).

### NF-κB Pathway

The over-activation of nuclear factor-κB (NF-κB) with a role in the inflammatory response, immune response and cell apoptosis, is associated with multifarious tumors (61). Previous studies have demonstrated that the activation of the NF-κB pathway blocked the degradation and promoted the transcription of Snail1 (62), subsequently facilitating the migration and invasion in breast, colorectal, gastric cancers, cholangiocarcinoma and malignant human keratinocyte (63–66).

## Regulation of Snail1 Expression by Ubiquitin-Proteasome Ststem

Ubiquitin mediates protein degradation *via* binding to lysine residues of the substrate proteins (67). It is highly conserved in eukaryotic cells and can also function as a signaling molecule to modulate protein function (68). Its eight residues including M1, K6, K11, K27, K29, K33, K48, and K63 are used as attachment sites to form polyubiquitin chains (69). The most abundant chain types are K48, which are usually degraded by the 26S proteasome (68).

### Degradation of SNAIL1 by UPS

Snail1 is an extremely unstable protein, β-TrCP1 was first reported to be involved in Snail1 ubiquitination *via* GSK-3β mediated phosphorylation of S96 and S100 residues on Snail1 (10). In contrast, Snail1 is ubiquitinated independently of GSK-3β phosphorylation by FBXL14 through K98, 137, and 146 residues (70). Interestingly, miR-27a can directly down-regulate the expression of FBXO45, resulting in reduced Snail1 degradation (71). In breast cancer, the S11 residue of Snail1 is phosphorylated by PKD1, which promotes the ubiquitination and degradation of Snail1 by FBXO11 (72, 73), while FBXO22 depends on GSK-3β (74). In addition, it has been reported that PPIL2, SPSB3 and TRIM21 are involved in ubiquitination and degradation of Snail1 (75–77). In gastric cancer, phosphorylation of Snail1 is required for the F-box domain of FBXO31 to function (78), FBXW7 inhibits metastasis in part by binding to Snail1 (79) and FBXL5 promotes poly-ubiquitination of Snail1 at K85, K146 and K234 residues (80, 81). In Non-small cell lung cancer, both β-TrCP2 and FBXW7 are absolutely implicated in ubiquitination and degradation of Snail1 (82, 83). In cervical cancer, HECTD1-mediated degradation of Snail1 occurs in the cytoplasm rather than in the nucleus (84). Finally, other E3 ligases such as TRIM50 and CHIP, are also involved in regulation of Snail1 in hepatocarcinoma and ovarian cancer, respectively (85, 86).

**Figure 2:**
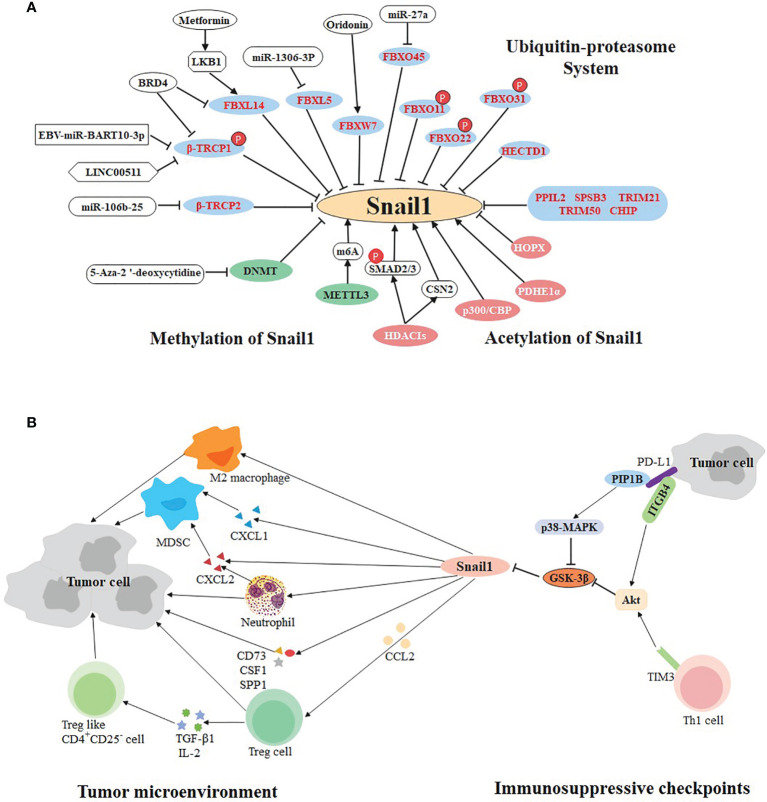
Multifaceted regulation of SNAIL1. **(A)** Ubiquitination, methylation, and acetylation regulate the expression of SNAIL1. The ubiquitin E3 ligases in the blue circle negatively regulate the expression of Snail1. Small molecule compounds or drugs directly or indirectly act on E3 ligase to promote or inhibit the expression of Snail1. Molecules in the green and pink circles participate in methylation and acetylation of Snail1, respectively. **(B)** Bidirectional regulation of SNAIL1 and tumor immune environment. Snail1 recruits immunosuppressive cells (including Treg, MDSCs, M2 macrophages, neutrophils, and Treg-like CD4+CD25- cells) to participate in the formation of tumor microenvironment via cytokines, chemokines and their receptors. In addition, immunosuppressive checkpoints PD-L1 and TIM-3 can regulate the expression of Snail1 through different molecular pathways.

So far, some molecular targets have been found based on the above E3 ligases, which are potential therapeutic targets. LINC00511 and EBV-miR-Bart10-3p both inhibit β-TrCP1 and prevent Snail1 degradation in triple negative breast cancer and nasopharyngeal carcinoma, respectively (87, 88). In non-small cell lung cancer, the expression of the β-TrCP2 is inhibited by miR-106b-25 (83), while FBXW7 agonist (Oridonin) contributes to the degradation of Snail1 (89). In hepatocellular carcinoma, miR-1306-3p directly targets FBXL5 to suppress Snail1 degradation (90). Likewise, miR-27a immediately down-regulate the expression of FBXO45 (91). Particularly, BRD4 identifies acetylated K146 and K187 on Snail1 in an acetylation-dependent manner to prevent its degradation by FBXL14 and β-TrCP1 in gastric cancer (92). Inversely, Metformin is beneficial to the expression of LKB1, thereby strengthening the capacity of FBXL14 in pancreatic cancer (93) **(** **Figure 2A** **)**.

### Stabilizing the Expression of SNAIL1 by DUBs

Ubiquitination is a reversible process and ubiquitin moieties are removed from polypeptides by deubiquitinases (DUBs) (94). Currently, plentiful DUBs are involved in the occurrence, progression, and drug resistance of cancer (95–97). In esophageal squamous cell carcinoma, OTUB1 inhibits the ubiquitination and degradation of SMAD2/3, leading to strengthen TGF-β signaling and stabilization of Snail1 expression (98, 99). Interestingly, USP26 is a specific deubiquitinase of Snail1 and significantly increases its stability by combining with the zinc finger domain at the Snail1, an essential region for its stability and nuclear localization (100, 101). In addition, EIF3H and PSMD14 have also been found to be involved in Snail1 deubiquitination (102, 103). In breast cancer, DUB3 couples on SNAG domain of Snail1 and inhibits ubiquitination of Snail1 mediated by FBXL14 and β-TrCP1 (104). Analogously, CSN2 removes the ubiquitination of Snail1 *via* disrupting its binding to GSK-3β and β-TrCP (62). In lung cancer, CSN5 and USP37 significantly stabilize the expression of Snail1. More importantly, USP37 is closely associated with increased mortality and metastasis rates (105, 106). In glioblastoma, USP3 also hydrolyzes FBXO11 or FBXW1-induced polyubiquitination chain on Snail1, resulting in increased aggressiveness and tumorigenicity (107). Similarly, high expression of OTUB1 in gliomas is associated with poor prognosis (108). In colorectal cancer, up-regulation of USP47 is mediated by SOX9, leading to an increase in Snail1 deubiquitination under hypoxia condition (109). In gastric cancer, USP29 enhanced the interaction between Snail1 and SCP1, causing both dephosphorylation and deubiquitination of Snail1 (110).

## Epigenetic Modification in Snail1 Regulation

Epigenetic abnormalities have been linked to many human diseases, including cancer (111, 112). Particularly, methylation and acetylation are involved in Snail1-mediated tumor metastasis **(** **Figure 2A** **)**.

### Methylation of SNAIL1

DNA methylation is an important mechanism of epigenetic gene regulation, which primarily occurs at CpG dinucleotide within gene promoters by a covalent modification of cytosine residues *via* DNA methyltransferase (DNMT) enzymes (113). It was previously reported that DNA methylation in the first intron region of Snail1 was negatively correlated with its transcription level, but its expression was increased when treated with DNMT inhibitor 5-Aza-2 ‘-deoxycytidine in trophoblast cells (114). Uniformly, the chromatin remodeling factor ARID2 represses EMT of hepatocellular carcinoma by recruiting DNMT1 to Snail1 promoter, which increases promoter methylation and inhibits its transcription (115). Recently, m6A RNA methylation is an emerging epigenetic modification, which has been associated with the progression of several cancers (116, 117). Interestingly, m6A is methylated by Methyltransferase-like 3 (METTL3) to accelerate Snail1 expression in HeLa cells (118), which is equivalent to indirect regulation of Snail1 by methylation.

### Acetylation of SNAIL1

Protein acetylation was originally discovered on histones in the nucleus and involved in gene transcription (119). Subsequently, non-histone proteins were increasingly found to also undergo acetylation (120). In nasopharyngeal carcinoma, the glucose metabolizing enzyme PDHE1α facilitates H3K9 acetylation on the Snail1 promoter to enhance cell motility and thereby drive cancer metastasis (121). Inversely, HOPX mediates epigenetic silencing of Snail1 transcription through the enhancement of histone H3K9 deacetylation in the Snail1 promoter (122). In lung cancer cells, p300 acetylates Snail1 at K187 (123), and CREB-binding protein (CBP) interacts with and acetylates Snail1 at K146 and K187, which prevents formation of the repressor complex (124). As mentioned above, BRD4 recognizes acetylated K146 and K187 on Snail1 to prevent it from being degraded by E3 ligases in gastric cancer (92).

At present, histone deacetylase inhibitors (HDACIs) are now emerging as a new class of anticancer agents (125, 126). However, HDACIs stabilize surprisingly Snail1 expression through several mechanisms in hepatocellular carcinoma: HDACIs up-regulate Snail1 at the transcriptional level by promoting SMAD2/3 phosphorylation and nuclear translocation (127). Posteriorly, HDACIs regulate the stabilization of Snail1 *via* up-regulating the expression of CSN2, which interacts with Snail1 to expose its acetylation site, leading to inhibit degradation of Snail1 *via* preventing its phosphorylation and ubiquitination (127). Coincidentally, this phenomenon was also observed in CNE2 cells (128). Accordingly, more cautions should be exercised in the usage of medicines such as HDACIs, as they may increase the risk of tumor metastasis.

## Bidirectional Regulation of Snail1 and Tumor Immune Environment in Tumor Progression

Tumorigenesis and progression are influenced by tumor microenvironment and controlled by the host immune system (129). In addition to malignant cells, adipocytes, fibroblasts, tumor vasculature, lymphocytes, dendritic cells, and cancer-associated fibroblasts are present in the tumor microenvironment (130). The last decade has witnessed dramatic advances in cancer treatment through immunotherapy such as immune checkpoints inhibitors, which are the most popular and promising treatment at present (131). Recently, the bidirectional regulation of immune checkpoints and EMT was uncovered *via* Snail1 **(** **Figure 2B** **)**.

### Immunosuppressive Checkpoints Regulate SNAIL1 Expression

So far, two immune checkpoint proteins PD-L1 and TIM-3 have been found to regulate Snail1 expression. PD-L1, which accumulates to high level on the surface of some tumor cells, can bind to PD-1 and induce T cells exhaustion, thereby mediating tumor immune escape and potentiating tumor progression (132, 133). Histochemical staining of 477 lung adenocarcinoma specimens showed a positive correlation between the expression of PD-L1 and Snail1 (134). Two studies showed that PD-L1 can inhibit GSK3β activity *via* binding to tyrosine phosphatase PTP1B or integrin β4 to activate p38-MAPK or Akt activity, respectively. Through this mechanism, PD-L1 can inhibit GSK3β-mediated phosphorylation, ubiquitination, and degradation of Snail1, thereby promoting EMT and the metastatic potential of breast cancer and cervical cancer (135, 136).

TIM-3 contains an immunoglobulin and a mucin-like domain and was originally identified as a receptor expressed on Th1 cells (137). The silencing of TIM-3 was accompanied by a decrease in Snail1 expression, indicating that TIM-3 may be involved in metastasis of osteosarcoma and hepatocellular carcinoma (138–140). Due to the lack of research in this aspect, it is only known that TIM-3 induces EMT to stimulate the metastasis of esophageal squamous cell carcinoma at least partly through the Akt/GSK-3β/Snail1 signaling pathway (141).

### The role of SNAIL1 in Tumor Immune Evasion

Increasing evidence suggests that Snail1 is also involved in immune escape from tumors, which can accelerate cancer metastasis. Previous research has reported the quantity of tumor-specific infiltrating lymphocytes and the systemic immune response increased *via* silencing Snail1 in melanoma (142), suggesting that Snail1 is visibly involved in tumor immunity. Firstly, Snail1 recruits CD4+FOXP3+Treg cells into the tumor microenvironment through C-C motif chemokine ligand 2 (CCL2) (143). In a mouse model of lung cancer, Snail1 was also found to increase intratumoral C-X-C chemokine ligand 2 (CXCL2) secretion and neutrophil infiltration (144). In ovarian cancer, Snail1 accelerates cancer progression *via* up-regulation of CXCL1 and CXCL2 as well as recruitment of myeloid-derived suppressor cells (MDSCs) (145), which plays a vital role in cancer immunosuppression, tumor angiogenesis, drug resistance and promotion of tumor metastasis (146, 147). In cholangiocarcinoma, Snail1 appears to produce immunosuppressive natural T-regulatory like CD4+CD25- cells, in part by mediating the T regulatory-inducible cytokines such as TGF-β1 and IL-2 (148). In addition, Snail1 induces M2 polarization of tumor-associated macrophages and facilitates tumor growth in head and neck cancer (149). A recent study showed that the high expression of Snail1 in mesenchymal tumor cell induces the expression of several cytokines (CD73, CSF1, SPP1), which collectively expedites the assembly of tumor immunosuppressive microenvironments (2). All these lines of evidence strongly confirm that Snail1 effectively promotes tumor cells to secrete chemokines or cytokines, which recruits various immunosuppressive cells to the tumor microenvironment and provides an appropriate environment for tumor metastasis.

## Summary and Future Perspectives

Physiologically, Snail1 participates in embryo implantation and initiation, wound healing, and cell survival (8, 150, 151). In addition, as we discussed above, Snail1 is a crucial target involved in tumor metastasis and immune escape, and can endow tumor cells with the characteristics of stem cells (5). What’s more, Snail1 overexpression was found to be a potential risk factor of neoplasm recurrence in various cancers, such as cutaneous squamous cell carcinoma, clear cell renal cell carcinoma, ameloblastic carcinoma, non-muscle-invasive bladder, colon and non-small-cell lung cancer (152–157). Consistent with tumor relapse, Snail1 overexpression also indicates poor prognosis in several types of cancers (158–161). Taken together, Snail1 can function as a biomarker to predict tumor relapse and patient prognosis.

Snail1 also hold critical role in cancer treatment, increasing evidence suggested that Snail1 is implicated in chemotherapy and radiotherapy resistance. For instance, silencing Snail1 was found to be beneficial in enhancing the sensitivity of gemcitabine therapy in pancreatic ductal carcinoma (162, 163) and increasing radiosensitivity in hypopharyngeal carcinoma (164). Furthermore, Snail1 contributes to the resistance of glioblastoma cells to temozolomide *via* the IL-6-STAT3-Snail1 pathway (165) and colorectal cancer cells to 5-fluorouracil by facilitating the expression of the ABCB1 resistance gene (166). In addition, Snail1 overexpression could induce tumor stem cell-like phenotype and generate chemotherapy resistance to oxaliplatin in colorectal cancer (167). Collectively, chemotherapy or radiotherapy combined with Snail1 inhibitors such as CYD19 (168), GN-25 (169) and Co (III)-Ebox (170) may be a promising therapeutic approach to combat tumors. At present, it has not yet been reported whether Snail1 is involved in immune checkpoint blockade. Due to knockdown of Snail1 decreases the infiltration of immunosuppressive cells in the tumor microenvironment, it is possible targeting Snail1 could enhance the anti-tumor effect. Accordingly, further development of novel Snail1 inhibitors and investigation of the safety of these compounds is urgently need for conquering cancer in future.

## Author Contributions

XT and XS were responsible for the primary review of literature, consolidation of information, and writing. LW and YL guided and supervised this study. All authors contributed to the article and approved the submitted version.

## Funding

This work was supported by National Natural Science Foundations of China (81802400 to YL, 81900199 and 81974465 to LW), China Postdoctoral Science Foundation (2020M670053 to YL), Hunan province natural science funds for Excellent Young Scholars (2019JJ30043 to LW) and the recruitment program for Huxiang talents (2019RS1009 to LW).

## Conflict of Interest

The authors declare that the research was conducted in the absence of any commercial or financial relationships that could be construed as a potential conflict of interest.

## Publisher’s Note

All claims expressed in this article are solely those of the authors and do not necessarily represent those of their affiliated organizations, or those of the publisher, the editors and the reviewers. Any product that may be evaluated in this article, or claim that may be made by its manufacturer, is not guaranteed or endorsed by the publisher.
